# Prognosis and complications of patients with primary gastrointestinal diffuse large B‐cell lymphoma: Development and validation of the systemic inflammation response index‐covered score

**DOI:** 10.1002/cam4.5733

**Published:** 2023-03-03

**Authors:** Yurou Chu, Yingyue Liu, Yujie Jiang, Xueling Ge, Dai Yuan, Mei Ding, Huiting Qu, Fang Liu, Xiangxiang Zhou, Xin Wang

**Affiliations:** ^1^ Department of Hematology, Shandong Provincial Hospital Shandong University Jinan China; ^2^ Department of Hematology Shandong Provincial Hospital Affiliated to Shandong First Medical University Jinan China; ^3^ Shandong Provincial Engineering Research Center of Lymphoma Jinan China; ^4^ Branch of National Clinical Research Center for Hematologic Diseases Jinan China; ^5^ National Clinical Research Center for Hematologic Diseases the First Affiliated Hospital of Soochow University Suzhou China; ^6^ Campbell Family Mental Health Research Institute, Centre for Addiction and Mental Health, Department of Psychiatry University of Toronto Toronto Ontario Canada

**Keywords:** gastrointestinal complications, model, primary gastrointestinal diffuse large B‐cell lymphoma, prognosis, systemic inflammation response index

## Abstract

**Background:**

This study aimed to evaluate the predictive value of systemic inflammation response index (SIRI) in primary gastrointestinal diffuse large B‐cell lymphoma (PGI‐DLBCL) patients and establish a highly discriminating risk prediction model.

**Methods:**

This retrospective analysis included 153 PGI‐DCBCL patients diagnosed between 2011 and 2021. These patients were divided into a training set (*n* = 102) and a validation set (*n* = 51). Univariate and multivariate Cox regression analyses were conducted to examine the significance of variables on overall survival (OS) and progression‐free survival (PFS). An inflammation‐covered score system was established according to the multivariate results.

**Results:**

The presence of high pretreatment SIRI (≥1.34, *p* < 0.001) was significantly associated with poorer survival and identified as an independent prognostic factor. Compared with NCCN‐IPI, the prognostic and discriminatory capability of the novel model SIRI‐PI showed a more precise high‐risk assessment with a higher area under the curve (AUC) (0.916 vs 0.835) and *C*‐index (0.912 vs 0.836) for OS in the training cohort, and similar results were obtained in the validation cohort. Moreover, SIRI‐PI also showed good discriminative power for efficacy assessment. This new model identified patients at risk of developing severe gastrointestinal complications following chemotherapy.

**Conclusions:**

The results of this analysis suggested that the pretreatment SIRI may be a potential candidate for identifying patients with a poor prognosis. And we established and validated a better‐performing clinical model, which facilitated the prognostic stratification of PGI‐DLBCL patients and can serve as a reference for clinical decision‐making.

## Graphical abstract

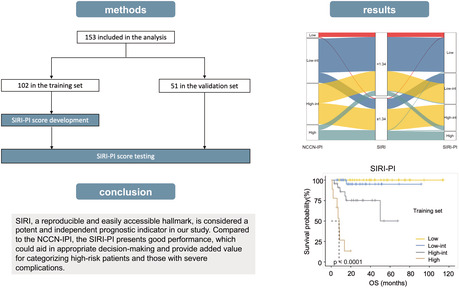

## 1 INTRODUCTION

In the modern era, primary gastrointestinal lymphoma comprises about 30%–40% of cases in primary extranodal lymphomas.^1^ And diffuse large B‐cell lymphoma is the most commonly occurring type in the gastrointestinal tract.^2^ The most frequently affected site of primary gastrointestinal diffuse large B‐cell lymphoma (PGI‐DLBCL) is the stomach, followed by the small intestine and ileocecal region.^3^, ^4^ To date, there is still no consensus on its optimal regimens. The current treatments for PGI‐DLBCL involve chemotherapy, radiotherapy, surgery, and double or triple combination therapies mentioned above.^5^ At present, R‐CHOP (rituximab, cyclophosphamide, doxorubicin, vincristine, and prednisone)‐like therapies represent the standard therapeutic protocol in the majority of patients, especially in people at advanced stages.^6^

Although PGI‐DLBCL is viewed as a potentially curable disease compared with other extranodal lymphoma types, several patients still present unfavorable outcomes and require early risk assessment.^7^ A multitude of gene‐based signatures and biochemical markers have been considered to improve the risk assessment, while those are often unavailable and expensive.^8^, ^9^, ^10^ Hence, it is critical to seek more accurate, feasible, and easily obtainable markers to distinguish patients who suffer a particularly aggressive course. Besides, clinical complications of PGI‐DLBCL could trigger specific management difficulties, such as perforation and massive bleeding. Specifically, severe complications in PGI‐DLBCL can result in delayed and complicated treatment, reduced quality of life, and even increased mortality.^11^ Currently, the role of the widely known score, the National Comprehensive Cancer Network (NCCN)‐International Prognostic Index (IPI), remains to be determined in the PGI‐DLBCL cohort. There is a need for new prognostic models incorporating simple and reliable prognostic factors for PGI‐DLBCL that can predict the likelihood of complications and stratify cases into clinically meaningful subsets.

Tumor‐associated inflammation is a landmark feature of tumor development and progression.^12^ In the clinical setting, various studies conveyed that the systemic inflammatory response may predict inferior outcomes in patients with different neoplasms, such as gastric malignancies,^13^ pancreatic cancer,^14^ leukemia,^15^ and Hodgkin's lymphoma.^16^ Recently, accumulating studies suggested that several inflammatory markers, including neutrophil to lymphocyte ratio (NLR), platelet to lymphocyte ratio (PLR), lymphocyte to monocyte ratio (LMR), and systemic immune inflammation index (SII), were prognostic factors in lymphoma.^16^, ^17^, ^18^, ^19^ Systemic inflammation response index (SIRI), a novel inflammatory marker estimated by the levels of neutrophils, monocytes, and lymphocytes, was demonstrated as an independent prognostic index in gastric cancer.^20^ In the meantime, mounting evidence showed that combined inflammatory scores uncovered a closer and more profound linkage between markers and cancer.^21^, ^22^, ^23^, ^24^, ^25^ Notwithstanding, the role of SIRI in PGI‐DLBCL patients was limited and underestimated.

Herein, we developed a simple and easily applicable score to predict prognosis, treatment efficacy, and posttreatment gastrointestinal complications (GICs) in patients with PGI‐DLBCL. By incorporating the SIRI and NCCN‐IPI, this predictive tool may help distinguish high‐risk patients and assist in implementing appropriate clinical decisions.

## 2 PATIENTS AND METHODS

### 2.1 Patients

We retrospectively evaluated the clinical characteristics and follow‐up data of 153 patients with PGI‐DLBCL (86 patients with gastric DLBCL and 67 patients with intestinal DLBCL) newly diagnosed at Shandong Provincial Hospital between January 2011 and February 2022. All patients recruited met the following criteria: (1) pathologically diagnosed as gastrointestinal DLBCL, (2) no previous chemotherapy or immunotherapy treatment, (3) no previous history of malignancy or immunosuppression, (4) receiving CHOP‐based therapies, and (5) with a complete series of clinical information and follow‐up data. In all cohorts, data included patients' age, gender, primary site, germinal center B‐cell‐like (GCB) versus non‐GCB, systemic B symptoms, Ann Arbor stage (I‐IV), Eastern Cooperative Oncology Group (ECOG) performance status (PS), involvement of extranodal sites, NCCN‐IPI, serum lactate dehydrogenase (LDH), Alb, prognostic nutritional index (PNI), β2‐microglobulin (β2‐MG), NLR, LMR, PLR, SII, and SIRI. All these laboratory parameters were extracted from serum ≤3 days before the first chemotherapy or immunochemotherapy treatment. Furthermore, a cohort of 8301 patients diagnosed with PGI‐DLBCL between 2000 and 2018 was obtained from the SEER database. Overall survival (OS), the primary endpoint, was defined as the period from diagnosis to death from any cause or the last follow‐up time. Progression‐free survival (PFS) was determined as the period from the diagnosis to the earliest death, disease recurrence, or disease progression.

### 2.2 Definition of SII, SIRI, PNI, and severe GICs

SII is described as (P × N)/L, where P, N, and L represent platelet, neutrophil, and lymphocyte counts, respectively. SIRI is calculated as neutrophil × monocyte/lymphocyte (N × M/L). PNI is estimated as 10 × serum albumin (g/dL) + 0.005 × total lymphocyte count/mm^3^.

Severe postchemotherapy GICs include gastrointestinal bleeding (GIB), obstruction (GIO), and perforation (GIP), which can be identified by clinical testing, imaging, or surgery. Specifically, severe GIB was outlined as a bleeding episode such as hematemesis, melena, and/or bloody stools.^26^ Also, occult bleeding requiring blood transfusion was taken into account. Concerning GIO, both partial and complete obstructions were considered, with no exhaust or passage of feces.^27^ GIP was diagnosed when free air was seen under the diaphragm on an abdominal X‐ray or CT scan, or when perforation was discovered during a laparotomy.^28^

### 2.3 Construction and validation of the prognostic model

The recruited patients were randomly assigned to a training cohort (*n* = 102) and a validation cohort (*n* = 51) based on a 2:1 ratio. The multivariate Cox proportional hazard regression analyzed clinical characteristics associated with survival, inflammatory markers, and the components of NCCN‐IPI. Owing to the multivariate results, a new multiparameter prognostic model was constructed by the integration of the NCCN‐IPI and SIRI. The prognostic performance and discriminatory power of two risk score models were measured by area under the curve (AUC), which was derived from the receiver operating characteristic (ROC) curve, and Harrell's *C*‐index. We also calculated the integrated discrimination improvement (IDI), the continuous version of the net reclassification improvement (NRI),^29^ and calibration curves to measure model performance when comparing risk prediction models.^30^

### 2.4 Statistical analysis

SPSS 25.0 and R software 4.1.2 were used to conduct the statistical analyses and draw figures. Clinical data were gathered using SEER*Stat software (version 8.3.6). Baseline clinical features were assessed by the Chi‐squared test or Fisher's exact test for categorical variables. The unpaired *t*‐test analyzed continuous variables if the data followed a normal distribution and had the same variance, otherwise, the Mann–Whitney *U* test compared them. ROC curve analysis estimated the optimal cutoff values for quantitative variables such as SIRI, SII, LMR, NLR, and PLR. The time‐dependent ROC curves of scores were created by the “timeROC” package in R, and forest plots were drawn by the “forestplot” package. In addition, IDI and NRI were evaluated by the “survIDINRI” package. Decision curve analysis (DCA) was used to assess the net clinical benefit of the models. Survival analyses were performed using Cox regression and the log‐rank test. If the log‐rank test was not applicable for some survival plots, we performed a landmark analysis. The weights were calculated in accordance with the respective *β* coefficients from the multivariable analysis.^31^ Median follow‐up time was measured by the reverse Kaplan–Meier method. We used restricted cubic splines to flexibly model and visualize the association between SIRI and severe postchemotherapy GICs. The two‐sided *p* < 0.05 was regarded as statistically significant, and hazard ratios (HRs) were estimated with 95% confidence intervals (95% CIs).

## 3 RESULTS

### 3.1 Baseline characteristics of patients

An initial cohort of 201 patients in our center and 8301 patients in the SEER database with PGI‐DLBCL were enrolled in this study. Table S1 shows the demographic and clinical characteristics of the SPH and SEER datasets. Regarding clinical features, such as sex, primary site, B symptoms, and surgery or not, there was no statistical difference between the two datasets. For the SEER cohort, the majority of patients were male (60.7%), almost the same as in our center (58.2%). We also observed that patients with gastric DLBCL were evenly distributed (52.8% vs 54.7%) in the two cohorts. Regarding treatment, relatively fewer patients underwent surgery (36.8% vs 43.0%) in the SEER database. Clinical characteristics, including race, age, and stage, showed significant differences across the different datasets. The SEER database showed a higher proportion of elderly patients and patients with stage I–II.

The flow chart in Figure S1 represents the patient selection procedure in our study. A total of 153 patients were involved in our study, including 86 patients with gastric DLBCL and 67 patients with intestinal DLBCL, with a median age of 56 years (range: 18–84 years). The baseline characteristics of these patients are shown in Table S2. There were 86 (56.2%) males and 67 (43.8%) females. The median follow‐up time was 32.0 months (95% CI: 24.8–39.2), ranging from 2 to 120 months. A total of 45 patients (29.3%) presented B symptoms at the initial assessment, 110 (71.9%) patients had stage III or IV disease, and 37 (24.2%) patients exhibited more than one extranodal involvement. Forty‐five patients (29.4%) showed ECOG PS with more than two scores. Serum LDH was discovered to be higher in 44 cases (28.8%). One hundred and fifteen (75.2%) patients received R‐CHOP‐based treatment, while 38 (24.8%) cases received CHOP‐like regimes. In the light of the NCCN‐IPI, seven cases (4.6%) were classified as low risk (NCCN‐IPI = 0–1), and most patients were identified as low‐intermediate risk (66 cases, 43.1%), ranging from 2 to 3 points, or intermediate‐high risk (59 cases, 38.6%). Besides, 21 patients (13.7%) were defined as high risk (NCCN‐IPI ≥6).

### 3.2 Optimal cutoff values of inflammatory indexes and relationship with OS and PFS

The predictive values of inflammatory indices (NLR, LMR, PLR, SII, and SIRI) were evaluated by the ROC curves (Figure S2). According to the 5‐year OS, the highest AUC exhibited here was uncovered for SIRI (0.822) (sensitivity 92.9%, specificity 71.6%), while the AUC for SII was 0.795 (sensitivity 71.4%, specificity 87.5%). The outcomes intensely favored SIRI instead of other inflammatory indicators, for it had the optimal discriminatory capacity shown both in short‐ and long‐term follow‐up (Figure S2). Then, the optimal cutoff values for the SIRI, SII, NLR, LMR, and PLR were 1.34, 1230, 4.75, 1.63, and 275, respectively. Among these 102 patients in the training cohort, the associations between SIRI and baseline clinical features of PGI‐DLBCL patients are exhibited in Table S3. In the two sets, elevated SIRI was observed in patients with laboratory parameters of decreased Alb, LMR, and PNI, together with increased LDH, NLR, PLR, and SII (all *p* < 0.05).

**FIGURE 1:**
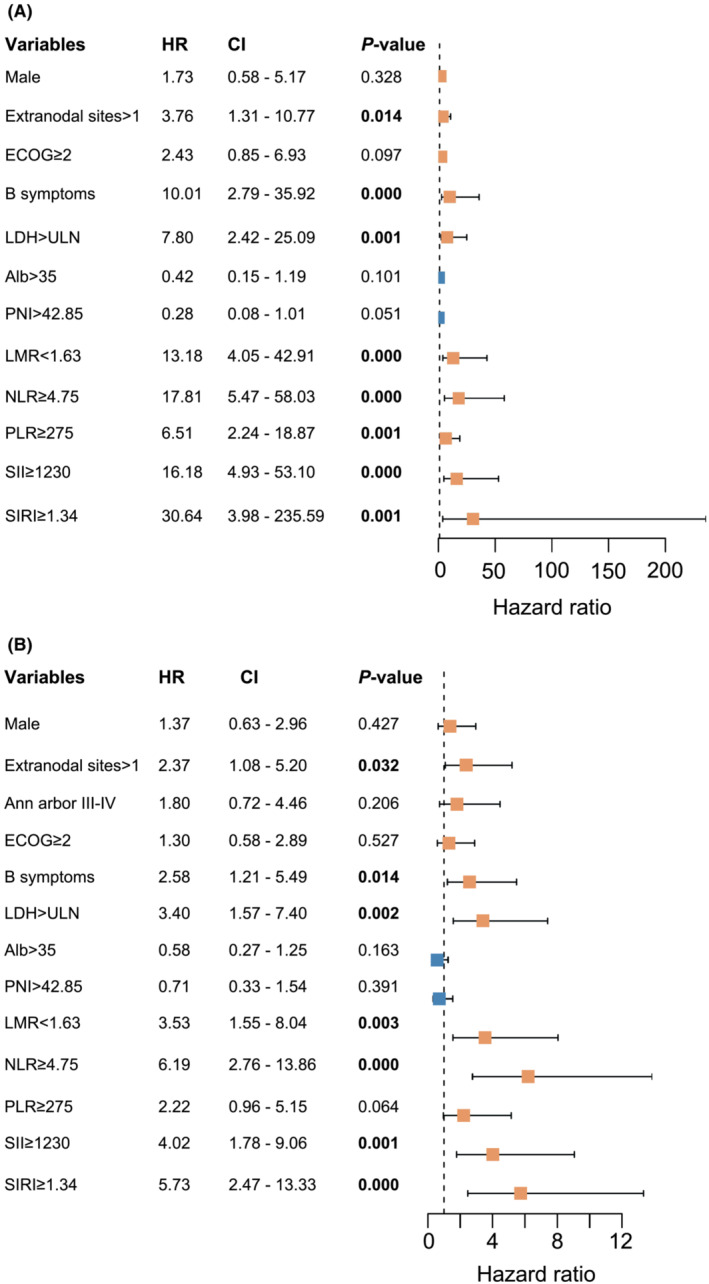
Forest plots of univariate analyses based on OS and PFS in the training set. (A) Univariate analysis of OS. (B) Univariate analysis of PFS. HR, hazard ratio; OS, overall survival; PFS, progression‐free survival; NLR, neutrophil to lymphocyte ratio; PLR, platelet to lymphocyte ratio; LMR, lymphocyte to monocyte ratio; SII, systemic immune inflammation index; SIRI, systemic inflammation response index; Alb, albumin; PNI, prognostic nutritional index; ECOG PS, Eastern Cooperative Oncology Group performance status; LDH, lactate dehydrogenase; CI, confidence interval.

In univariate regression analysis, the significant variables for OS and PFS comprised extranodal sites, LDH, B symptoms, NLR, LMR, SII, and SIRI (Figure 1). According to the multivariate Cox regression, associations between variables in OS and PFS were accessible (Table 1). Cox regression analysis revealed three negative prognostic factors for OS, including SIRI ≥ 1.34 (HR: 14.147, 95% CI: 1.368–146.292; *p* = 0.026), B symptoms (HR: 18.789, 95% CI:4.363–80.911; *p* = 0.000), and LDH > ULN (HR: 6.754, 95% CI: 1.607–28.397; *p* = 0.009). Moreover, SIRI ≥1.34 (HR: 2.811, 95% CI: 1.002–6.072; *p* = 0.050), B symptoms (HR: 2.881, 95% CI: 1.321–6.280; *p* = 0.008), and LDH > ULN (HR: 2.452, 95% CI: 1.028–5.850; *p* = 0.043) emerged as the indicators for considerably inferior PFS times.

**TABLE 1:** Multivariate analyses of OS and PFS.

Variables	OS		PFS	
	HR (95% CI)	*p*‐value	HR (95% CI)	*p*‐value
LDH > ULN	6.754 (1.607–28.397)	**0.009**	2.452 (1.028–5.850)	**0.043**
B symptoms	18.789 (4.363–80.911)	**0.000**	2.881 (1.321–6.280)	**0.008**
SIRI ≥ 1.34	14.147 (1.368–146.292)	**0.026**	2.811 (1.002–6.072)	**0.050**
NLR ≥ 4.75	3.156 (0.824–12.087)	0.094	2.262 (0.843–6.072)	0.105

*Note*: Bold indicates statistical significant value (*p* < 0.05).

Abbreviations: CI, confidence interval; HR, hazard ratio; LDH, lactate dehydrogenase; NLR, neutrophil to lymphocyte ratio; OS, overall survival; PFS, progression‐free survival; SIRI, systemic inflammation response index; ULN, upper limits of normal.

### 3.3 Development and validation of the SIRI‐PI prognostic score

A high level of SIRI was negatively and remarkably correlated with the OS and PFS of PGI‐DLBCL patients, owing to the multivariate analyses. Combining SIRI into a risk model (NCCN‐IPI) allowed us to predict clinical outcomes effectively and could enrich the current stratification. The novel model (SIRI‐PI) may suggest greater predictive accuracy than the NCCN‐IPI alone. Patients with SIRI ≥1.34 were allocated two points as a risk factor calculated in terms of the *β* coefficients compared with the effect of an elevated LDH level (>ULN) in the multivariate analysis of OS. This established an integrated scoring model with a maximum of 10 points (Table S4).

**FIGURE 2:**
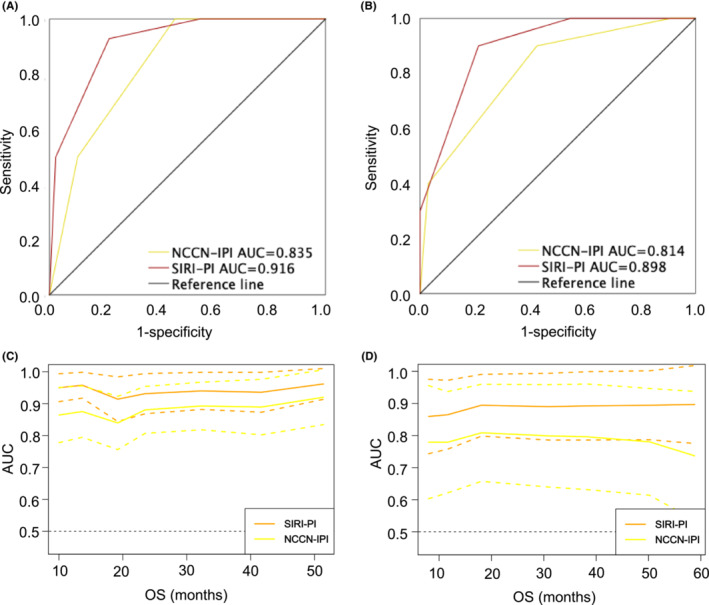
Validation of the NCCN‐IPI and SIRI‐PI for predicting OS in the training and validation sets. (A) ROC validation of OS in the training set. (B) ROC validation of OS in the validation set. (C) Time‐dependent ROC curves of OS in the training set. (D) Time‐dependent ROC curves of OS in the validation set. OS, overall survival; NCCN‐IPI, National Comprehensive Cancer Network‐International Prognostic Index; SIRI‐PI, prognostic index including the National Comprehensive Cancer Network‐International Prognostic Index and systemic inflammation response index.

Given the even distribution of clinical factors in the derivation and validation groups (Table 2), favorable discrimination was found in both cohorts. The AUC (0.916 vs 0.835) and *C*‐index (0.912 vs 0.836) were superior based on the SIRI‐PI in the training set, indicative of good discrimination (Figure 2A). According to the validation cohort, SIRI‐PI also surpassed the NCCN‐IPI with superior AUC (0.898 vs 0.814) and *C*‐index (0.845 vs 0.766) in discrimination (Figure 2B). To observe the changes in AUC more intuitively among these models during the follow‐up period, we also generated time‐dependent ROC curves to compare the prognostic accuracy of the NCCN‐IPI and SIRI‐PI. The time‐dependent ROC curves showed that SIRI‐PI was consistently superior to those of NCCN‐IPI throughout the observation period in both the derivation and validation groups (Figure 2C,D).

**TABLE 2:** Therapeutic efficacy according to SIRI‐PI score in the entire cohort.

	SIRI‐PI Low/low‐intermediate (*n* = 77)	SIRI‐PI High/high‐intermediate (*n* = 36)	*p‐*value
Radiologic response			
Complete/partial response	57 (74.0%)	17 (47.2%)	0.003
Stable disease	10 (13.0%)	4 (11.1%)	
Progressive disease	10 (13.0%)	15 (41.7%)	
Disease control			
Yes (CR/PR/SD)	67 (87.0%)	21 (58.3%)	0.001
No (PD)	10 (13.0%)	15 (41.7%)	

Abbreviations: CR, complete response; OS, overall survival; PD, progressive disease; PR, partial response; SD, stable disease; SIRI‐PI, prognostic index including the National Comprehensive Cancer Network‐International Prognostic Index and systemic inflammation response index.

**FIGURE 3:**
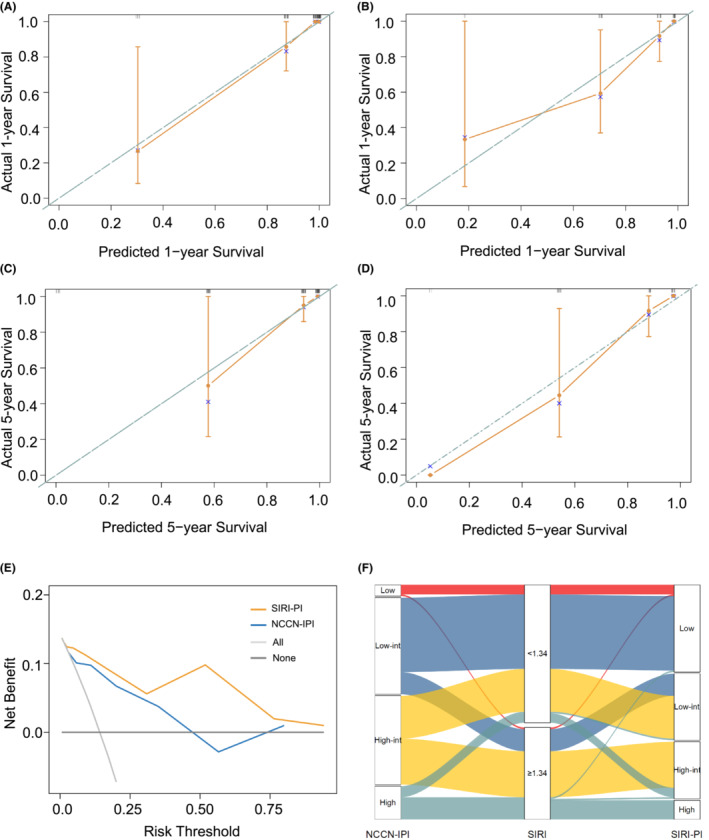
The calibration curves and decision curve analysis of the SIRI‐PI model and an alluvial plot shows the association of SIRI‐PI with other risk factors. (A) The calibration curve for 1‐year OS in the training set. (B) The calibration curve for 1‐year OS in the validation set. (C) The calibration curve for 5‐year OS in the training set. (D) The calibration curve for 5‐year OS in the validation set. (E) The decision curve analysis of the SIRI‐PI model at median OS time in the training set. (F) An alluvial plot shows the association of SIRI‐PI with other risk factors in the entire cohort. The dashed diagonal lines in the calibration curves represent ideal calibration plots. Low‐int, low‐intermediate; High‐int, high‐intermediate; NCCN‐IPI, National Comprehensive Cancer Network‐International Prognostic Index; SIRI, systemic inflammation response index; SIRI‐PI, prognostic index including the National Comprehensive Cancer Network‐International Prognostic Index and systemic inflammation response index.

**FIGURE 4:**
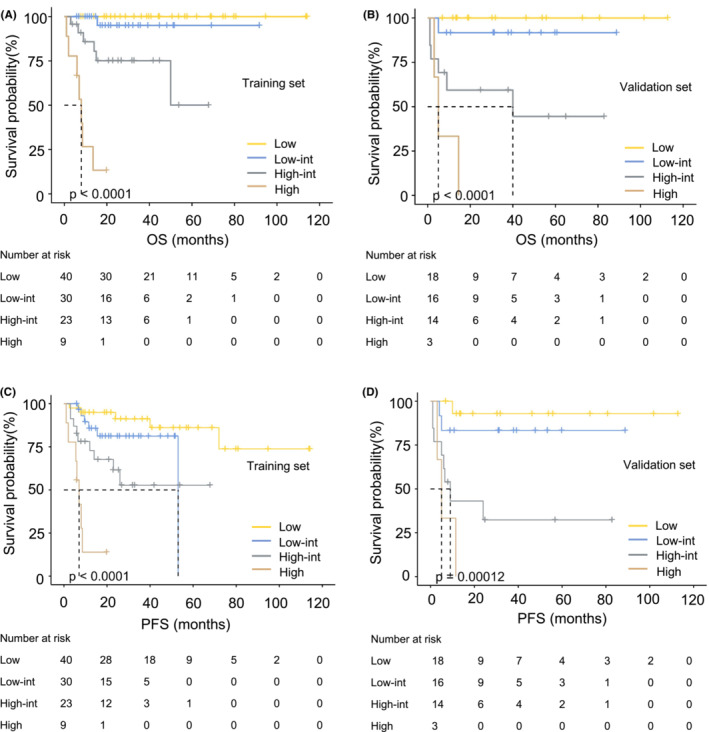
Kaplan–Meier analyses of PGI‐DLBCL patients stratified by SIRI‐PI. (A) Kaplan–Meier curves of OS in the training set. (B) Kaplan–Meier curves of OS in the validation set. (C) Kaplan–Meier curves of PFS in the training set. (D) Kaplan–Meier curves of PFS in the validation set. Significant differences in OS and PFS were observed between the risk groups (*p* < 0.001, log‐rank test, or landmark analysis, for each cohort). OS, overall survival; PFS, progression‐free survival; Low‐int, low‐intermediate; High‐int, high‐intermediate; NCCN‐IPI, National Comprehensive Cancer Network‐International Prognostic Index; SIRI, systemic inflammation response index; SIRI‐PI, prognostic index including the National Comprehensive Cancer Network‐International Prognostic Index and systemic inflammation response index.

The new model was well calibrated, as the predicted and observed survival of the primary outcome at 1 and 5 years after diagnosis are shown in Figure 3A–D, identifying a reliable predictive capability of the SIRI‐PI. Moreover, DCA described a higher net clinical benefit from the SIRI‐PI than the NCCN‐IPI in the development cohort (Figure 3E, Figure S3). The alluvial plot visually represents the relationship between SIRI‐PI, NCCN‐IPI, and SIRI in patients with PGI‐DLBCL in the entire cohort (Figure 3F). In the alluvial plot, a high‐risk group of SIRI‐PI consisted of patients with a high NCCN‐IPI score (≥6) and a high level of SIRI (≥1.34). The survival curves were well separated, and the validation scoring system showed good discriminatory capacity in the cohorts in line with OS and PFS (Figure 4). Considering the low OS rates observed in Figure 4, the new risk model showed that patients in the high‐risk group of SIRI‐PI faced unfavorable outcomes. A pooled population of 153 patients was divided into four risk groups according to the SIRI‐PI (low: 0–3; low‐intermediate: 4–5; high‐intermediate: 6–7; and high: ≥8). In comparison to the NCCN‐IPI, which only had one laboratory parameter, the new multiparameter prognostic model was able to enhance risk classification, particularly in classifying patients at high risk.

To further demonstrate the improvement of our prediction tool in its discriminative capability, the improvement of the SIRI‐PI over the NCCN‐IPI was assessed by calculating the IDI and NRI. According to OS, the prediction performance of two models at 5 years in the development group was shown as 0.260 (95% CI: 0.140–0.355) for IDI and 0.723 (95% CI: 0.480–0.936) for NRI, respectively. The IDI was 0.26, indicating a 26% relative improvement in the discrimination slope with the addition of the SIRI.

### 3.4 Predictive value of SIRI‐PI score in therapeutic efficacy

According to the follow‐up imaging, radiologic tumor response was assessed in 113 patients after six cycles of treatment, corresponding to 73.9% of the study population (*n* = 153). Overall, the objective response rate in PGI‐DLBCL patients was 65.5%, with 49 (43.4%) complete responses (CR) and 25 (22.1%) partial responses (PR). Fourteen (12.4%) patients had stable disease (SD), and 25 (22.1%) patients had progressive disease (PD) at the first radiographic assessment. According to the SIRI‐PI model, 113 patients were divided into two groups: the low/low‐intermediate cohort and the high/high‐intermediate cohort. Concerning the efficacy outcomes, both cohorts presented distinct and disparate efficacy outcomes in terms of radiation tumor response (*p* = 0.003) and disease control (*p* = 0.001), suggesting good predictive power for clinical applications when assessing efficacy (Table 2).

### 3.5 Predictive value of SIRI‐PI score in severe GICs

**FIGURE 5:**
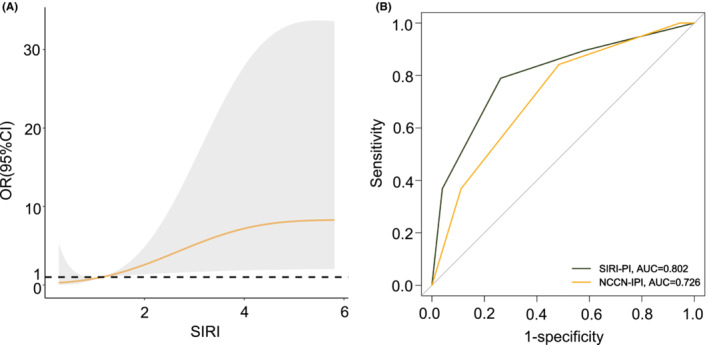
Association of SIRI (as a continuous factor) with the odds of developing severe gastrointestinal complications (GICs) and ROC validation of SIRI‐PI in the entire cohort. (A) Association of SIRI with the odds of developing severe GICs. The solid line represents the log hazard ratio. The shaded area is the 95% confidence interval. (B) ROC validation of SIRI‐PI in the entire cohort. OR, odd ratio; ROC; receiver operating characteristic; CI, confidence interval; SIRI‐PI, prognostic index including the National Comprehensive Cancer Network‐International Prognostic Index and systemic inflammation response index.

Severe gastrointestinal events after chemotherapy occurred in 11.8% (18/153) of patients, including GIO in 7.8% (12/153), GIB in 3.3% (5/153), and only one patient (0.7%) was recorded for GIP (Table S5). In Figure 5A, with the increase in SIRI level, the probability of severe complications increased significantly, indicating the predicted value of SIRI in severe GICs. Figure 5B highlights the good performance of SIRI‐PI (AUC: 0.802) in predicting patients at high risk of suffering from complications. Severe complications occurred mainly during the first four cycle of chemotherapy, requiring additional surgical intervention.

## 4 DISCUSSION

In this study, we performed a retrospective analysis emphasizing the prognostic value of SIRI in PGI‐DLBCL and investigating the possible prognostic model built for PGI‐DLBCL. There is an overlapping impact between inflammatory indicators, and the precise prognostic function of these indicators remains unclear. Hence, it is necessary to assess the profound significance of one specific biomarker that can better reflect survival outcomes and complications in PGI‐DLBCL. Within the article, compared with several inflammation‐based factors, SIRI was the most predominant prognostic marker based on ROC curve analysis and was retained for further estimation.

To date, numerous studies have corroborated that tumor progression is triggered by the inherent properties of malignant cells plus systemic inflammatory responses. A series of surveys have been performed to explore the prognostic value of diverse systemic inflammatory indexes, including SIRI, SII, NLR, LMR, PLR, PNI, and Alb, in several neoplasms, albeit the potential mechanisms are not fully elucidated.^17^, ^32^ The prognostic significance of SII has been analyzed in cancers arising from the stomach or intestine with a broad range of optimal cutoff values, while SIRI has not been widely assessed.^23^, ^33^ Although each of the SII, NLR, LMR, PLR, PNI, and Alb indexes confers a vital prognostic impact in various malignancies. SIRI, an innovative biomarker that considerably correlates with systemic inflammation, appears to have a more potent and consistent prognostic benefit in some cancer types.^34^, ^35^, ^36^

SIRI concurrently integrates all three cells (neutrophils, lymphocytes, and monocytes) extracted from serum and, thereby, allows better assessment of inflammatory responses associated with tumorigenesis and progression. Likewise, contrasted with single‐cell components, the consolidated SIRI is less likely to be affected in diverse clinical settings: infections, impaired liver function, fluid retention, and dehydration. Some studies demonstrated that platelets were a helpful factor in predicting dismal results and could improve the predictive ability of IPI in patients with DLBCL.^37^ However, these results did not apply to our PGI‐DLBCL patients. As a result, the outcome measurement of objectives in this study judged by SIRI instead of SII can ascribe to the limited predictive capacity of platelets among patients. Strengthened by these findings, there is a solid rationale to conduct this present exploration in PGI‐DLBCL patients despite the lack of similarly designed studies.

Presently, the NCCN‐IPI, as an enhanced IPI score, has been widely utilized for the stratification of DLBCL. Nonetheless, due to a lack of consideration for the role that adaptive immunity and the tumor microenvironment play in the pathogenesis of lymphoma, this stratification tool cannot well differentiate high‐risk populations when taking rituximab‐based therapies. The model established by combining the SIRI and NCCN‐IPI can predict the survival of PGI‐DLBCL patients more effectively, as it could yield a better ROC curve and *C*‐index than the NCCN‐IPI. Namely, the prognostic model delivered superior prognostication and risk evaluation of oncologic outcomes. Also, our model is related to survival and achieving disease control (response or stabilization). As a result, it may define patients with a poor prognosis and predict poorly responding patients, allowing for more appropriate and efficient treatments for those with PGI‐BLBCL. It is prudent to interpret the results of our study according to the newly established model, for it is supposed to be of additional value in guiding the appropriate treatment for PGI‐DLBCL patients rather than replacing NCCN‐IPI in practice.

Perforation, massive bleeding, and obstruction are potentially life‐threatening complications of lymphomas involving the gastrointestinal tract and result in considerable morbidity, which requires timely identification and treatment.^28^ Once severe complications occur, an array of adverse outcomes may follow, including infections, prolonged hospitalization, complications of wound healing, and delays in follow‐up treatment. As DLBCL is considered the most common type of lymphoma associated with perforation, physicians should consider the risk of perforation after initial treatment.^28^ Obstruction, the most frequently observed severe clinical complication in our study, was associated with a significantly reduced quality of life and the delaying of chemotherapy. Consequently, identifying the risk of severe GICs after chemotherapy can help clinicians pay more attention and adopt optimal treatment to reduce the incidence of fatal GICs. In addition, the prompt treatment also provided opportunities to complete the remaining systemic chemotherapy.

Given the nature of retrospective and single‐center analysis, the findings of this study were accompanied by a few limitations. First, we might have been subject to potential selection bias in data collection. Second, according to the limited number of enrolled patients and the deficiency of external validation of this model, our analyses should rather be considered exploratory than confirmatory. Third, there might be overlap between high SIRI and other high‐risk factors, such as B symptoms and higher LDH. Further analyses should take into account these high‐risk characteristics collectively since they may be associated with the patients' inflammatory state. Lastly, as a single‐center study, it is anticipated that our results demand to be confirmed in a larger cohort of patients to demonstrate the generalizability of our model and be explored in distinct clinical settings. Despite differences in patient race, there was no significant difference in the distribution of baseline data such as sex, primary site, B symptoms, and whether surgery was performed or not in our database and the SEER database, which may provide the possibility for the application of this prognostic model. Nevertheless, the prognostic model needs further verification due to a higher portion of patients with stage III‐IV and younger patients in our research.

## 5 CONCLUSION

Overall, SIRI, a reproducible and easily accessible hallmark, is considered a potent and independent prognostic indicator in our study. Compared to the NCCN‐IPI, the SIRI‐PI presents good performance, which could aid in appropriate decision‐making and provide added value for categorizing high‐risk patients and those with severe complications.

## AUTHOR CONTRIBUTIONS

**Yurou Chu:** Data curation (lead); formal analysis (lead); investigation (equal); writing – original draft (lead). **Yingyue Liu:** Data curation (equal). **Yujie Jiang:** Data curation (equal). **Xueling Ge:** Data curation (equal). **Dai Yuan:** Formal analysis (supporting). **Mei Ding:** Data curation (equal). **Huiting Qu:** Formal analysis (supporting). **Fang Liu:** Writing – review and editing (supporting). **Xiangxiang Zhou:** Writing – review and editing (equal). **Xin Wang:** Writing – review and editing (equal).

## FUNDING INFORMATION

This study was supported by National Natural Science Foundation (nos. 82270200, 82170189, 82070203, 81800194, 81770210); Key Research and Development Program of Shandong Province (2018CXGC1213); Development Project of Youth Innovation Teams in Colleges and Universities of Shandong Province (2020KJL006); China Postdoctoral Science Foundation (nos. 2021T140422, 2020M672103); Translational Research Grant of NCRCH (nos.2021WWB02, 2020ZKMB01); Shandong Provincial Natural Science Foundation (ZR2021YQ51); Technology Development Project of Jinan City (no. 202134034); Taishan Scholars Program of Shandong Province; Shandong Provincial Engineering Research Center of Lymphoma; and Academic Promotion Programme of Shandong First Medical University (nos. 2019QL018, 2020RC006).

## CONFLICT OF INTEREST STATEMENT

The authors have no relevant conflicts.

## ETHICAL APPROVAL AND PATIENT CONSENT

The study was conducted in accordance with the Declaration of Helsinki. This study was approved by the Medical Ethical Committee of Shandong Provincial Hospital Affiliated to Shandong University. And informed consent was waived because of its retrospective nature.

## Supporting information


Data S1.

## Data Availability

The datasets are available from the corresponding author on reasonable request.
