# Supplementary material for: Prognosis and complications of patients with primary gastrointestinal diffuse large B‐cell lymphoma: Development and validation of the systemic inflammation response index‐covered score
Source: Cancer Med. 2023 Mar 3;12(8):9570–82. doi: 10.1002/cam4.5733 (PMC10166949; doi:10.1002/cam4.5733)
Supplement: Supplementary file 1 — Data S1. [file CAM4-12-9570-s001.docx]

**Inflammation-covered Score Predicts Prognosis and Complications of Patients with Primary Gastrointestinal Diffuse Large B-Cell Lymphoma**

**Supplement information**

**Contents**

Supplementary Table 1. Patient characteristics of SPH database and SEER database ............................ 2

Supplementary Table 2. Patient characteristics according to the training and validation cohort........ 3

Supplementary Table 3. Patient characteristics in the training cohort according to the SIRI≥1.34 versus <1.34 ......................................................................................................................................................... 4

Supplementary Table 4. The detailed description of SIRI-PI....................................................................6

Supplementary Table 5. The severe post-chemotherapy gastrointestinal complications in the whole cohort.........................................................................................................................................................7

Supplementary Figure 1. Patients’ enrolment and model development ...................................................8

Supplementary Figure 2. The predictive ability of inflammatory markers was compared by receiver operating characteristic curve for 1 and 5 years .......................................................................................9

Supplementary Figure 3. The decision curve analyses of the SIRI-PI model for 1-year and 5-year OS in the training set..........................................................................................................................................10

**Table S1.** **Patient characteristics of SPH database and SEER database.**

| characteristics | SPH database (n=201) | SEER database (n=8301) | *P*-value |
| --- | --- | --- | --- |
| Age at diagnosis, years |  |  | 0.000 |
| Median | 57 | 67 |  |
| Range | 6-91 | 3~85+ |  |
| ≤60 | 117(59.7%) | 2625(31.6%) |  |
| >60 | 84(40.3%) | 5676(68.4%) |  |
| Sex |  |  | 0.468 |
| Male | 117(58.2%) | 5042(60.7%) |  |
| Female | 84(41.8%) | 329(39.3%) |  |
| Race |  |  | 0.000 |
| White | 0 | 6793(81.8%) |  |
| Black | 0 | 588(7.1%) |  |
| Other | 201(100.0%) | 920(11.1%) |  |
| Ann Arbor stage |  |  | 0.000 |
| I-II | 78(38.8%) | 5733(69.1%) |  |
| III-IV | 123(61.2%) | 2568(30.9%) |  |
| Primary site |  |  | 0.589 |
| Stomach | 110(54.7%) | 4383(52.8%) |  |
| Intestine | 90(45.3%) | 3918(47.2%) |  |
| Surgery |  |  |  |
| Yes | 80(43.0%) | 3060(36.8%) | 0.085 |
| No | 106(57.0%) | 5244(63.2%) |  |
| B symptom |  |  | 0.389 |
| Yes | 61(30.3%) | 878(10.6%) |  |
| No | 140(69.7%) | 1762(21.2%) |  |
| Unknown | 0 | 5673(68.2%) |  |

Abbreviations: SPH: Shandong Provincial Hospital; SEER: Surveillance, Epidemiology, and End Results.

**Table S2** Patient characteristics according to the training and validation cohort.

| Variables | Total cohort (n=153) | Primary cohort (n=102) | Validation cohort (n=51) | *P*-value |
| --- | --- | --- | --- | --- |
| Sex |  |  |  |  |
| Male | 86(56.2%) | 56(54.9%) | 30(58.8%) | 0.645 |
| Female | 67(43.8%) | 46(45.1%) | 21(41.2%) |  |
| Age |  |  |  |  |
| Age>60 | 61(39.9%) | 43(42.2%) | 18(35.3%) | 0.414 |
| Age≤60 | 92(60.1%) | 59(57.8%) | 33(64.7%) |  |
| ECOG PS |  |  |  |  |
| ≥2 | 45(29.4%) | 32(31.4%) | 13(25.5%) | 0.452 |
| <2 | 108(70.6%) | 70(68.6%) | 38(74.5%) |  |
| Ann Arbor stage |  |  |  |  |
| I-II | 43(28.1%) | 32(31.4%) | 11(21.6%) | 0.203 |
| III-IV | 110(71.9%) | 70(68.6%) | 40(78.4%) |  |
| Extranodal site |  |  |  |  |
| >1 | 37(24.2%) | 25(24.5%) | 12(23.5%) | 0.894 |
| ≤1 | 116(75.8%) | 77(75.5%) | 39(76.5%) |  |
| COO |  |  |  |  |
| GCB | 55(36.0%) | 39(38.2%) | 16(31.4%) | 0.382 |
| Non-GCB | 77(50.3%) | 49(48.1%) | 28(54.9%) |  |
| Not evaluable | 21(13.7%) | 14(13.7%) | 14(13.7%) |  |
| B symptom |  |  |  |  |
| Present | 45(29.4%) | 31(30.4%) | 14(27.5%) | 0.707 |
| Absent | 108(70.6%) | 71(69.6%) | 37(72.5%) |  |
| Rituximab |  |  |  |  |
| Yes | 115(75.2%) | 74(72.5%) | 41(80.4%) | 0.290 |
| No | 38(24.8%) | 28(27.5%) | 10(19.6%) |  |
| Surgery |  |  |  |  |
| Yes | 48(31.4%) | 35(34.3%) | 13(25.5%) | 0.268 |
| No | 105(68.6%) | 67(65.7%) | 38(74.5%) |  |
| LDH |  |  |  |  |
| >ULN | 44(28.9%) | 29(28.7%) | 15(39.4%) | 0.929 |
| ≤ULN | 108(71.1%) | 72(71.3%) | 36(70.6%) |  |
| SIRI |  |  |  |  |
| ≥1.34 | 62(39.9%) | 38(37.3%) | 23(45.1%) | 0.350 |
| <1.34 | 92(60.1%) | 64(62.7%) | 28(54.9%) |  |
| NCCN-IPI |  |  |  |  |
| low | 7(4.6%) | 4(3.9%) | 3(5.9%) | 0.734 |
| Low-intermediate | 66(43.1%) | 44(43.1%) | 22(43.1%) |  |
| High-intermediate | 59(38.6%) | 38(37.3%) | 21(41.2%) |  |
| High | 21(13.7%) | 16(15.7%) | 5(9.8%) |  |

Abbreviations: ECOG PS, Eastern Cooperative Oncology Group performance status; Alb, albumin; PNI, prognostic nutritional index; GCB, germinal center B-cell-like; LDH, lactate dehydrogenase; SIRI, systemic inflammation response index; NCCN-IPI National Comprehensive

Cancer Network-International Prognostic Index.

**Table S3** Patient characteristics in the training cohort according to the SIRI≥1.34 versus <1.34.

| Variables | Training cohort (n=102) | SIRI≥ 1.34 (n=38) | SIRI<1.34 (n=64) | *p*-value |
| --- | --- | --- | --- | --- |
| Sex |  |  |  |  |
| Male | 56(54.9%) | 19(50.0%) | 37(57.8%) | 0.443 |
| Female | 46(45.1%) | 19(50.0%) | 27(42.4%) |  |
| Age |  |  |  |  |
| Age>60 | 43(42.2%) | 20(52.6%) | 23(35.9%) | 0.099 |
| Age≤60 | 59(57.8%) | 18(47.4%) | 41(64.1%) |  |
| ECOG PS |  |  |  |  |
| ≥2 | 32(31.4%) | 17(44.7%) | 15(23.4%) | 0.025 |
| <2 | 70(68.8%) | 21(55.3%) | 49(76.6%) |  |
| Ann Arbor stage |  |  |  |  |
| 1-2 | 32(31.4%) | 6(15.8%) | 26(40.6%) | 0.009 |
| 3-4 | 70(68.8%) | 32(84.2%) | 38(59.4%) |  |
| Extranodal site |  |  |  |  |
| >1 | 25(24.5%) | 14(32.8%) | 11(17.2%) | 0.026 |
| ≤1 | 77(75.5%) | 24(67.2%) | 53(82.8%) |  |
| COO |  |  |  |  |
| GCB | 38(37.3%) | 11(28.9%) | 28(43.8%) | 0.156 |
| Non-GCB | 49(48.0%) | 21(55.3%) | 28(43.8%) |  |
| Not evaluable | 14(13.7%) | 6(15.8%) | 8(12.5%) |  |
| B symptom |  |  |  |  |
| Present | 31(30.4%) | 14(36.8%) | 17(26.6%) | 0.275 |
| Absent | 71(69.6%) | 24(63.2%) | 47(73.4%) |  |
| Rituximab |  |  |  |  |
| Yes | 74(72.5%) | 27(71.1%) | 47(73.4%) | 0.794 |
| No | 28(27.5%) | 11(28.9%) | 17(26.6%) |  |
| Surgery |  |  |  |  |
| Yes | 35(34.3%) | 9(23.7%) | 26(40.6%) | 0.081 |
| No | 67(65.7%) | 29(37.3%) | 38(59.4%) |  |
| LDH |  |  |  |  |
| >ULN | 31(30.4%) | 23(39.5%) | 8(12.5%) | <0.001 |
| ≤ULN | 71(69.6%) | 15(60.5%) | 56(87.5%) |  |
| Alb |  |  |  |  |
| ≥35 | 71(69.6%) | 20(52.6%) | 51(79.7%) | 0.004 |
| <35 | 31(30.4%) | 18(47.4%) | 13(20.3%) |  |
| PNI |  |  |  |  |
| ≥42.85 | 48(47.1%) | 10(26.3%) | 38(59.4%) | 0.001 |
| <42.85 | 54(52.9%) | 28(73.7%) | 26(40.6%) |  |
| LMR |  |  |  |  |
| ≥1.63 | 84(82.4%) | 22(57.9%) | 62(96.9%) | <0.001 |
| <1.63 | 18(17.6%) | 16(42.1%) | 2(3.1%) |  |
| NLR |  |  |  |  |
| ≥4.75 | 18(17.6%) | 18(47.4%) | 0(0%) | <0.001 |
| <4.75 | 84(82.4%) | 20(52.6%) | 64(100%) |  |
| PLR |  |  |  |  |
| ≥275 | 22(21.4%) | 17(44.7%) | 5(7.8%) | <0.001 |
| <275 | 80(78.4%) | 21(55.3%) | 59(92.2%) |  |
| SII |  |  |  |  |
| ≥1230 | 21(20.6%) | 21(55.3%) | 0(0%) | <0.001 |
| <1230 | 81(79.4%) | 17(44.7%) | 64(100%) |  |

Abbreviations: ECOG PS, Eastern Cooperative Oncology Group performance status; Alb, albumin; PNI, prognostic nutritional index; GCB, germinal center B-cell-like; LDH, lactate dehydrogenase; SIRI, systemic inflammation response index; SII, systemic immune inflammation index; NLR, neutrophil to lymphocyte ratio; PLR, platelet to lymphocyte ratio; LMR, lymphocyte to monocyte ratio.

**Table S4 The detailed description of SIRI-PI.**

| **SIRI-PI** |  |  |  |
| --- | --- | --- | --- |
| **Variables** | **Score** | **Risk group** | **Score** |
| Age, years | 1 | Low | 0-3 |
| >40 to ≤60 | 2 | Low-intermediate | 4-5 |
| >60 to <75 | 3 | High-intermediate | 6-7 |
| ≥75 |  | High | ≥8 |
| LDH |  |  |  |
| >ULN to ≤3ULN | 1 |  |  |
| >3ULN | 2 |  |  |
| Ann Arbor stage III-IV | 1 |  |  |
| Extranodal disease | 1 |  |  |
| ECOG PS ≥2 | 1 |  |  |
| SIRI≥1.34 | 2 |  |  |

**Table S5 The** **severe post-chemotherapy gastrointestinal complications in the whole cohort.**

| **Complications** | **Stomach (n=86)** | **Intestinal (n=67)** | ***p*-value** |
| --- | --- | --- | --- |
| Gastrointestinal bleeding | 8 | 4 | 0.447 |
| Gastrointestinal obstruction | 2 | 3 | 0.654 |
| Gastrointestinal perforation | 0 | 1 | 0.438 |
| Total | 10 | 8 |  |

**Figure S1 Patients’ enrolment and model development.**


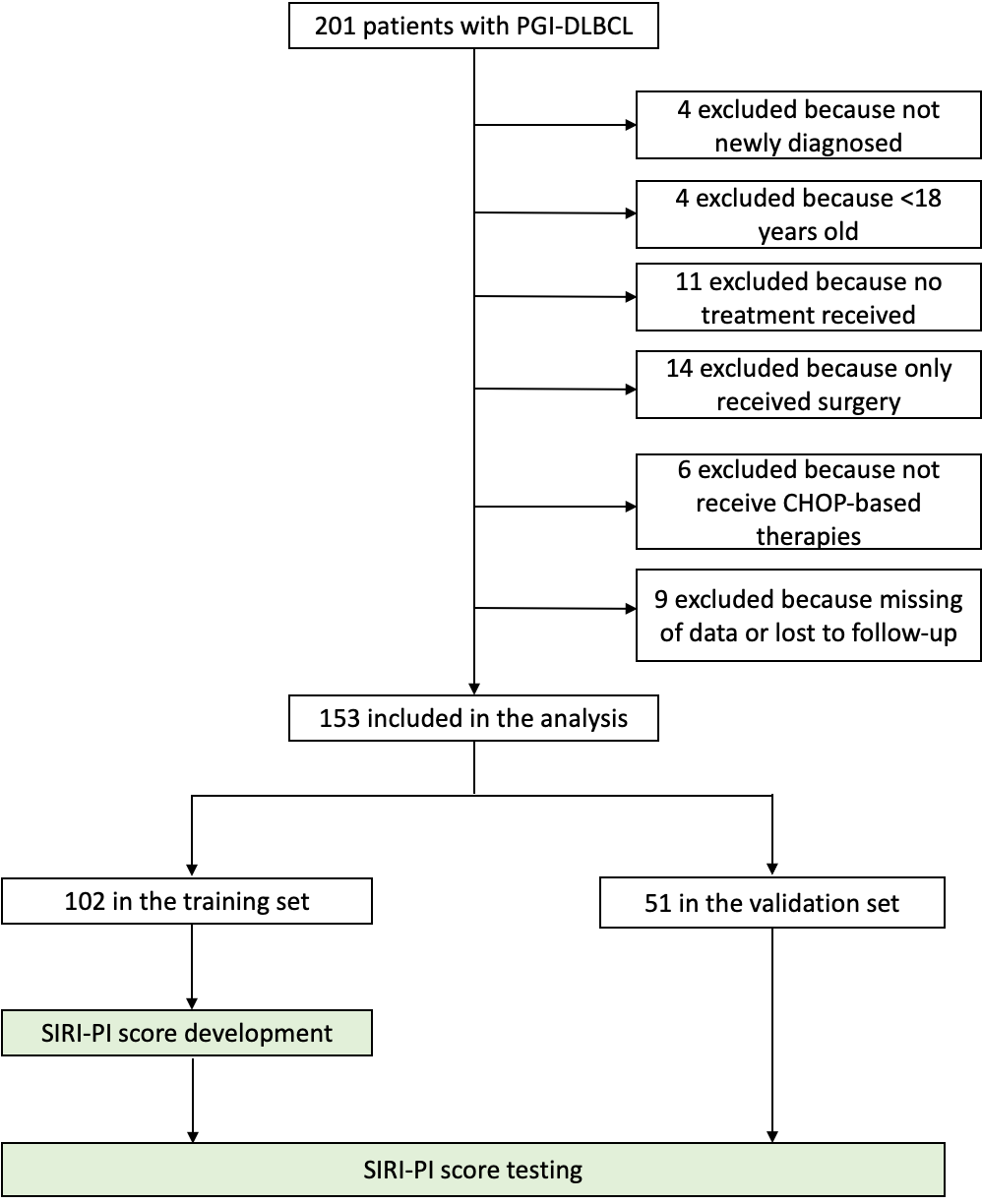


Abbreviations: SIRI-PI, prognostic index including the National Comprehensive Cancer Network-International Prognostic Index and systemic inflammation response index.

**Figure S2 The predictive ability of inflammatory markers was compared by receiver operating characteristic curve for 1 and 5 years.**


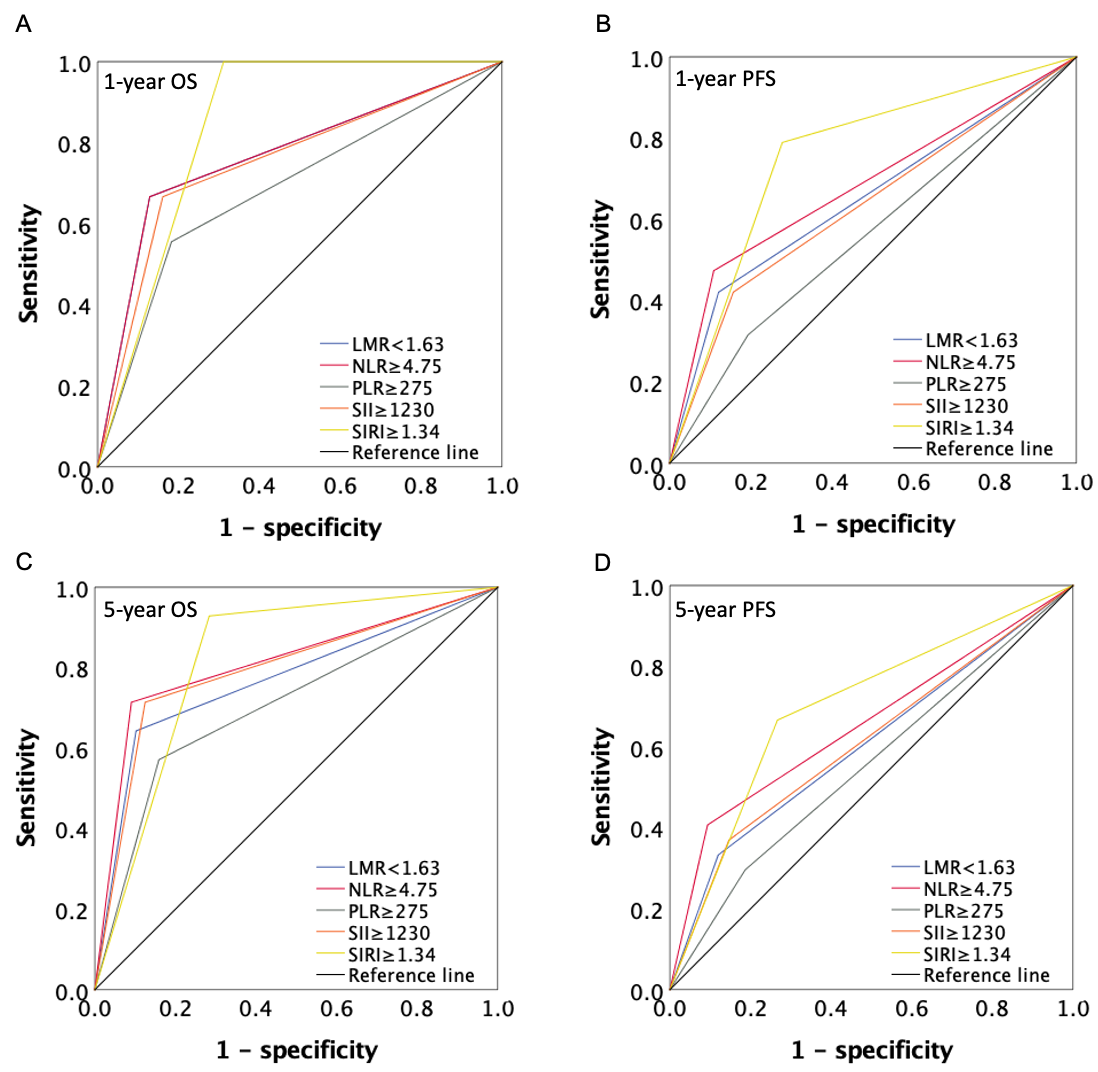


Abbreviations: NLR, neutrophil to lymphocyte ratio; PLR, platelet to lymphocyte ratio; LMR, lymphocyte to monocyte ratio; SII, systemic immune inflammation index; SIRI, systemic inflammation response index.

**Figure S3 The decision curve analyses of the SIRI-PI model for 1-year and 5-year OS in the training set.**


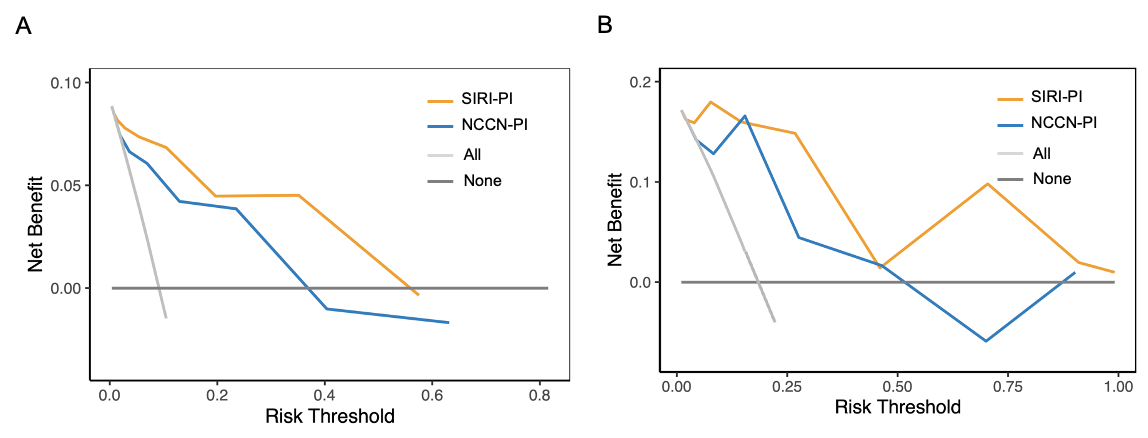


A: the decision curve analysis of 1-year OS in the training set. B: the decision curve analysis of 5-year OS in the training set.
